# Nintinol Self-Expandable Metallic Stenting in Management of Malignant Obstructive Jaundice: A Case Series

**Published:** 2010-03-01

**Authors:** Hossein Ghanaati, Kavous Firouznia, Seyed Mehran Vaziri Bozorg, Ahmad Reza Ghasemi Esfe, Marzieh Motevallei, Mohammad Reza Abedini, Saed Rahmat Sadeghi

**Affiliations:** 1 Advanced Diagnostic and Interventional Radiology Research Center (ADIR), Imam Khomeini Hospital,Tehran University of Medical Sciences, Tehran, Iran; 2 Department of Radiology, Shahid Rajai Hospital, Iran University of Medical Sciences, Tehran, Iran

**Keywords:** Malignant Jaundice, Biliary Metal Stents, Percutaneous Biliary Stenting

## Abstract

**Background and Aims:**

Palliation therapy is the only available therapeutic method for most patients with tumor-induced obstructive jaundice. Metallic stents are now performed percutaneously as an alternative route to the endoscopic approach. It is widely accepted because of its safety, good patency rate, and minimal invasiveness. This study was designed to evaluate the long-term results of metallic self-expandable stent insertion in patients with malignant stenosis of the biliary tree.

**Methods:**

It is a longitudinal study of patients with percutaneously biliary stenting from September 2005 to March 2009. The patients had unresectable malignant biliary obstruction with unsuccessful endoscopic stenting and access. Percutaneous transhepatic cholangiogram performed after adequate local anesthesia, under sonographic or fluoroscopic guidance. Stenting or balloon dilation was performed through the hydrophilic guide wire. Among 50 patients, 45 stents were placed in biliary tree stenosis sites. Patients’ follow-up was during the first, second, third, and then the sixth month after insertion of biliary stents. Stent patency was considered successful in our patients, when there were no lab results or sonographic appearance of biliary tree obstruction.

**Results:**

10(20%) patients’ stent placement treatment failed because of unsuccessful technical procedure. The stenosis of biliary tract was complete and passage of guide wire was not possible through the tumor growth. 6 (15 %) patients with successful stent placements died within one month (mean, 22 days). Total serum bilirubin resolved to below 1.5 mg/dl within 30 days for 36 (90%) patients with successful stent placements. Early complications not leading to death occurred in 28% of cases. The mean survival time for all patients who underwent stent placement was 140 days (16-420days). The mean patency rate for all stents was 147 days.

**Conclusions:**

Percutaneous biliary stenting is a safe procedure with few technical complications and a high success rate of palliation for patients with malignant biliary jaundice. Early complications are mostly managed conservatively and death is mainly due to systemic effects of the malignant disease.

## Introduction

Malignant obstructive jaundice is a poor prognostic disease with important consequences for the patient’s lifestyle and survival. Most of the patients will die during the first six months after diagnosis.

There is no curative therapy for the disease,but stenting and drainage of the biliary tree have been proven as a safe method with few technical complications. Percutaneous transhepatic insertion of metallic stents is now a reliable method for palliation of patients with obstructive malignant jaundice, which is superior to plastic endoprosthesis when we consider the aspects of patency rate, quality of life, complication rate, and the cost of procedure [1][2].

In this study we decided to evaluate the long-term results of metallic self-expandable stent insertion in patients with malignant obstructive stenosis of the biliary tree. We observed the complications, the technical and clinical successfulness of the procedure, and the follow-up of patients referred to Imaging Medical Center as a tertiary center for palliation therapy.

## Material and Methods

This is a longitudinal study of 50 patients with unresectable malignant biliary jaundice who were referred to our center for palliation therapy using nitinol self-expandable metallic stents.

All patients were evaluated at the Imaging Medical Center (Imam Khomeini Hospital), and selected from a database of patients between September 2005 and March 2009.

The patients attending our center had unresectable malignant biliary obstruction; therefore, endoscopic retrograde cholangiopancreatography (ERCP) was not successful or possible and endoscopic stenting was not performed because of lack of endoscopic access.

The level of obstruction was proximal in 40 (80%) and distal in 10 (20%) patients.

Among 50 patients, two radiologists placed 45 stents into the biliary tree stenosis sites. This was done using the percutaneous route and transhepatic cholangiogram. The procedure was performed after adequate local anesthesia, using a 22 g Chiba needle to access a dilated biliary duct in the right and/or left lobe of the liver, under sonographic or fluoroscopic guidance. A 0.35 mm Guide wire was then passed towards the porta hepatis and a tract was dilated with a dilator (8F). A 5F catheter was then passed over the 0.35mm guide wire up to the obstruction site in the biliary duct. Next a 0.35mm hydrophilic guide wire was passed across the stenosis and into the common bile duct (CBD) and duodenum. Stenting or balloon dilation was performed through the hydrophilic guide wire and the tract was embolized with GelFoam (Gelita Medical) or patient’s clotted blood, at the end of the procedure, to prevent hemorrhaging and bile leakage.

**Figure 1:**
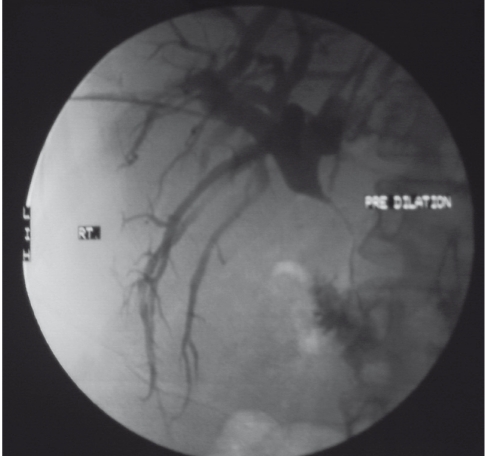
Percutaneous transhepatic cholangiogram of a patient with cholangiocarcinoma shows a severe stenosis of common bile duct

**Figure 2:**
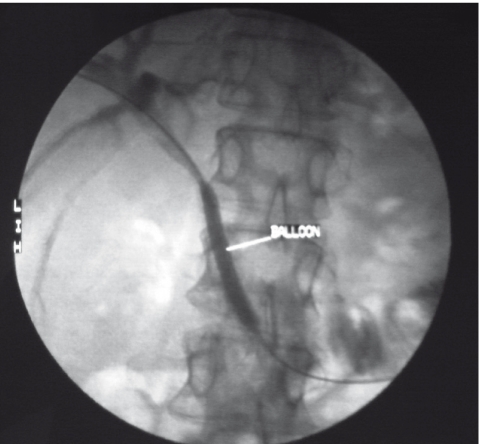
Same patient performing balloon dilation of the obstruction before stenting.

**Figure 3.**
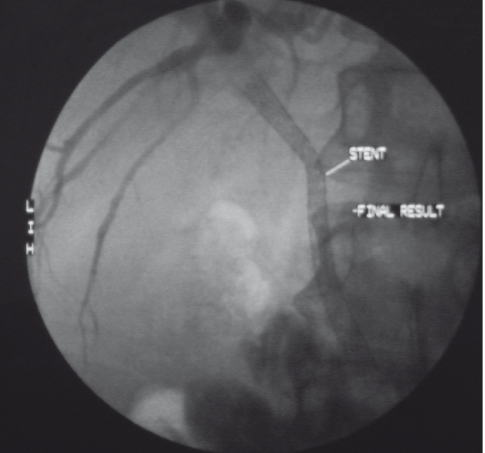
A) The situation immediately after placement of a metallic self-expanding biliary stent across the stenosis in the CBD.

Balloon dilation of the strictures in right or left hepatic ducts was performed before stenting in 14 (35%) of the patients with balloons (Boston Scientific, Galway, Irland) of 8mm diameters (Fig 1Fig 2fig 3afig 3b)

**B):**
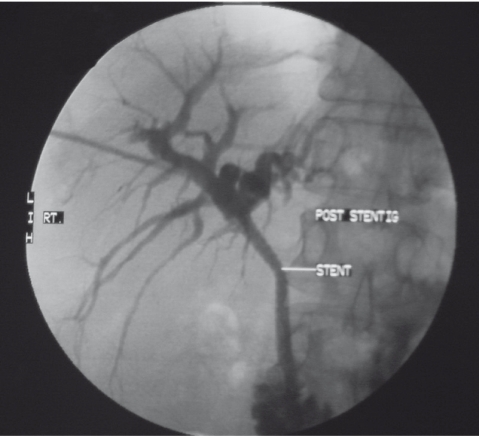
Final result showing fully expanded CBD stent.

Twenty-six (65%) patients underwent stent placement without predilation. A total of 45 selfexpandable nitinol metallic stents (Cook Medicals, USA) were used in our patients, and 5 patients needed two stents for sufficient bile duct drainage due to obstruction of common bile duct bifurcation and involvement of right and left hepatic ducts. 6-8mm was the diameter of the expanded stents and the length was 8-10 mm.

Among the 50 patients who were candidates for biliary stenting, 15 (30%) had undergone percutaneous transhepatic cholangiogram (PTC) and drainage before stent placement, with the range of 7 to 21 days.

Patient’s follow-up took place during the first,second, third, and then the sixth month after insertion of biliary stents. Death was the end point for follow-up in this study

Stent patency was considered successful in our patients, when there were no lab results or sonographic appearance of biliary tree obstruction. In the case of recurrent jaundice, CT and/or sonographic examinations were performed to detect restenosis or other complications.

The institutional radiological research division of the Tehran University of Medical Sciences has approved this study.

A general physician who was in contact with patients, by phone if necessary, recorded laboratory and clinical examinations.

Patient’s survival and stent patency were calculated and descriptive statistics (number and percentage for categorical variables, and number, mean, and standard deviation for continuous variables) were obtained using SPSS 16 software.

## Results

50 patients (32 males) were included in this study with the mean age being 69. They were referred to our center with primary diagnosis of either cholangiocarcinoma (25, 50%), pancreatic carcinoma (20, 40%), or gallbladder cancer (5, 10%).

Ten (20%) patients failed to treat by stent placement because of unsuccessful technical procedure. The stenosis of biliary tract was completed and passage of guide wire was not possible through the tumor growth. Six patients whose stent insertion failed had proximal biliary ducts obstruction, and four had severe distal stenosis. Six (15 %) patients with successful stent placement died within one month (mean, 22 days) of treatment.

The most important cause of death during this period was primary advanced disease and associated poor conditions (four patients). Other causes were cardio-pulmonary arrest because of sepsis, which was seen in two cases.

Total serum bilirubin resolved to below 1.5 mg/dl in36 (90%) patients with successful stent placement within 30 days. 10% of patients had no significant change in their serum bilirubin level. These patients with unchanged serum bilirubin all died within 40days (16-40days) of stent insertion.

Early complications not leading to death occurred in 28% of cases. Complications included mild free fluid in the abdomen (27%), pain and nausa (45 %), cholangitis (9%), and hemobilia (18%), which were recorded during the first 30 days of post-procedure follow up. None of the above complications needed surgical or interventional treatment.

**Figure 4:**
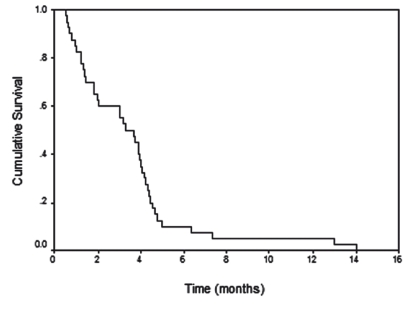
Survival curve for all patients.

The median survival time for all patients who underwent stent placement was 3.3 months (95% confidence interval [CI] = 2.27-4.33 months). 15%  died within 30 days, 26% during the second month of follow up, 49% within 3 to 5 months and 9% of our cases lived more than 6 months, with a maximum survival period of 371days (Fig 4).

From the total of 45 stents placed in biliary ducts, 37 (82%) stents were patent at the time of the patient’s death. Five (11%) stents need reintervention (ballooning and restenting), and 3 stents were totally occluded, therefore reintervention was unsuccessful. All of the patients who needed reintervention because of reobstruction of their stents lived more than 90 days.

All of the stents were patent at the time of the third month follow up. The mean patency rate for all stents was 147 days.

**Table 1:** Results of biliary stent placement

**Stent Insertion^a^**	
No. of Successful Cases	40 out of 50
Technical Success Rate (%)	80 %
**Biliary Drainage**	
No. of Successful Cases	36 out of 40
Clinical Success Rate (%)	90 %
**Early Complications**	11(28 %)
Pain and Nausa	5 (45 %)
Mild Free Fluid	3 (28 %)
Hemobilia	2 (18 %)
Cholangitis	1 (9 %)
Pancreatitis	0 (0 %)
Cholecystitis	0 (0 %)
**Late Complications**	
Tumor in Growth / Restenosis	8 (18 %)
**Follow Up**	
30 Day Mortality Rate	6 (15 %)
Patency Rate (mean)	4.9 months (147 days)
Mean Survival Time	4.6 months (140 days)
Median Survival Time	3.3 months (99 days)

^a^ Abbreviation: No. (%)

## Discussion

Percutaneous transhepatic biliary stenting for palliation of malignant obstructive jaundice has been considered a safe and well-established technique [1][2]. It has now been proven that metallic stent placement is superior to endoscopic plastic stent insertion [3][4]. In our study, we attempted to evaluate the technical success rate, complications and the patient’s outcome. It is a new procedure in our country and such academic reports can be used to help future improvements in this field.

Technical success rate in this study was 80% and early complication rate was 28%. The results in most other metallic wall stent reports have shown the technical success rate to be more than 90% (75- 100%) and early complication rate from 7 to 35% [1][2][5][6][7]. The cause and severity of the obstruction in each study are very different and it is difficult to compare the results, but as an explanation we think the main causes for lower technical success rate is the severity of the obstruction and the delay in patient’s referral time from clinicians; stent and procedure costs also might be another reason.

None of the complications in 30 days after stent insertion (without death) were significant enough to need further surgical or interventional treatments.

A 30 day mortality rate in our study was 15%; it falls within the reported range of 2-34% [7][8][9][10]. None of the early deaths occurred because of technical reasons, as systemic effects of malignancy were the main cause of death in this period.

The stent obstruction rate was 18% in our patients and is comparable to other studies with the late complication rate range (from stent failure) of 15 to 31 % [11][12]. Eighty-two percent of the patients died because of advanced disease and before stent occlusion happened.

Ninety percent of our patient’s serum bilirubin fell to normal limits during the first 30 days of stent placement [2][11][12]. They improved in clinical jaundice and pruritus; their clinical situation became clearly better, especially for those with expected survival rates of more than a month. They did not have to hold the external drainage bag and recuperated their appetite. However, it is unlikely whether the procedure had any advantages for patients who died within 30 days or not.

The most common late procedure-related complication was re-obstruction of the stents in patients who lived more than 90 days, similar to other studies [2], although it seems inevitable, it can be treated by re-intervention, at least in the majority of the cases. The results of multiple studies confirm that we could reach technical success rates of more than 90%. Most of the complications can be treated conservatively and the mortality rate of the procedure is significantly low (< 2%) in large series of surveys. In our study we had no procedure related mortalities [2]. All of the above could be a good reason for the increased usage of metallic stent placement for palliation of malignant biliary jaundice.

It seems that pre-procedure patient selection should be considered an important factor for biliary stent insertion, especially when poor condition suggests uncertainty for surviving more than a month. Who will be the best and benefit more from supportive care, and who will take more advantage from stent placement as a palliative treatment? We need to study more about the pre-procedure patient’s condition as there is a lack of adequate references in this area.
